# Early diagnosis of *Mycoplasma pneumoniae* pneumonia: Development and validation of a predictive model based on clinical examination indicators

**DOI:** 10.1371/journal.pone.0359335

**Published:** 2026-10-05

**Authors:** Yongwei Fan, Jingrun Zhou, Zhaoxi Wang, Huaqin Pan, Jiarui Zhang, Ling Wang, Guqin Zhang

**Affiliations:** 1 Department of Respiratory and Critical Care Medicine, Zhongnan Hospital of Wuhan University, Wuhan, Hubei, China; 2 The Respiratory Department of the Second Affiliated Hospital of Hainan Medical University, Haikou, Hainan, China; 3 Zhongnan Hospital of Wuhan University, Institute of Hepatobiliary Diseases of Wuhan University, Transplantation Intensive Care Unit, Transplant Center of Wuhan University, Hubei Key Laboratory of Medical Technology on Transplantation, Wuhan, Hubei, China; 4 Department of Critical Care Medicine, Zhongnan Hospital of Wuhan University, Clinical Research Center of Hubei Critical Care Medicine, Wuhan, Hubei, China; Children's National Hospital, George Washington University, UNITED STATES OF AMERICA

## Abstract

**Background:**

*Mycoplasma pneumoniae* accounts for a significant proportion of pathogens responsible for community-acquired pneumonia (CAP). Identifying *Mycoplasma pneumoniae* pneumonia (MPP) is challenging but essential for guiding early and appropriate antibiotic use.

**Methods:**

We developed a predictive model using data from hospitalized CAP patients diagnosed with either MPP or bacterial pneumonia between January and December 2023. Logistic regression and receiver operating characteristic (ROC) curve analyses were performed to explore associations between clinical features (age, symptoms, laboratory findings) and the causative pathogens, and then a prediction model was developed based on the results. The model’s clinical utility was assessed in an independent validation cohort comprising CAP patients who met the same inclusion/exclusion criteria in our hospital from 01/01/2024–30/06/2024.

**Results:**

A total of 244 patients were diagnosed with CAP, including 117 with MPP and 127 with bacterial pneumonia. Our analysis revealed that patients with MPP were younger (37.00 years vs. 61.00 years), had procalcitonin levels mostly within the normal range, exhibited higher serum albumin levels (40.68 g/L vs. 36.34g/L), and higher levels of complement C1q (209.30 mg/L vs. 169.55 mg/L) compared to patients with bacterial pneumonia. In contrast, thrombin time was relatively prolonged in patients with bacterial pneumonia (14.00 s vs. 13.10 s). Based on these findings, we developed a clinical prediction model with strong discriminatory performance for early differentiation between MPP and bacterial pneumonia with an area under the curve (AUC) of 0.879 (95% CI, 0.833–0.925). Temporal validation demonstrated the model’s stability and good predictive performance (AUC: 0.922 vs. derivation 0.879).

**Conclusions:**

Our study showed that age, serum albumin, complement C1q level, thrombin time, and normal-range procalcitonin levels were highly predictive of MPP. This study provides a predictive model for early differentiation between MPP and bacterial pneumonia to aid in timely clinical decision-making.

## Introduction

Community-acquired pneumonia (CAP) is a lower respiratory tract infection occurring outside of hospital settings. Epidemiological data indicate that it remains a major cause of hospitalization and mortality worldwide [1,2]. According to the Global Burden of Disease, Injury, and Risk Factors Study, emergency department visits for pneumonia averaged over 30% of the U.S. population aged 15 and older in 2021 [3]. In the pre-COVID-19 period, U.S. CAP cases were predominantly caused by bacteria and atypical pathogens, with *Streptococcus pneumoniae* and *M. pneumoniae* being the two most prevalent, according to the Global Burden of Disease Study 2016 [4,5]. However, pneumonias caused by these two most common pathogens are often clinically indistinguishable [6], which poses a significant challenge for physicians and then selecting appropriate antibiotics in early differential diagnosis.

Penicillins or cephalosporins are recommended as first-line empiric treatments for CAP in the 2019 guidelines from the American Thoracic Society and the Infectious Diseases Society of America [7]. Similar recommendations have been incorporated in Spanish Society of Pulmonology and Thoracic Surgery (SEPAR) Guidelines [8]. However, due to the absence of a cell wall, *Mycoplasma pneumoniae* exhibits intrinsic resistance to both β-lactam and cephalosporins antibiotics. Therefore, current empirical treatment in guidelines may provide inadequate coverage for *Mycoplasma pneumoniae* [7,9]*.* Moreover, delayed pathogen-directed antibiotics to MPP may cause the occurrence of severe MPP, even potentially progress to life-threatening manifestations [10]. Hence, the early identification of potential pathogens, especially for *Mycoplasma pneumoniae* and selection of appropriate antibiotics are crucial for improving clinical outcomes.

Current diagnostic approaches for MPP are mainly based on culture, serological, or molecular techniques, each with inherent limitations. According to the 2016 Chinese guidelines, two methods are accepted as definitive diagnostic criteria for MPP [9]: First, positive culture results from qualified respiratory specimens (e.g., sputum or alveolar lavage fluid, etc.) [9]. However, due to the prolonged growth cycle of *M. pneumoniae* and a low positive culture rate, the practical utility of this method is limited in clinical practice [11–13]. Next, in serology, the confirmation of *M. pneumoniae* infection requires demonstration of a fourfold or greater change in *M. pneumoniae*-specific antibody titers between the acute and convalescent phases [9]. This requirement limits the utility for early diagnosis of MPP because of the delayed antibody response. In addition to these methods, nasopharyngeal swab nucleic acid testing and IgM antibody assays currently represent the two widely utilized clinical approaches for *M. pneumoniae* diagnosis. However, both testing methods may also yield false-negative results due to factors such as inadequate sample collection or delayed antibody production, ultimately leading to missed diagnoses [14,15]. This highlights the need for alternative diagnostic markers to identify MPP patients and to support timely antibiotic decisions, therefore reducing disease duration and improving outcomes [16]. This study aims to develop a statistical model based on the analysis of routinely collected clinical data to enable early identification between MPP and bacterial pneumonia, thereby guiding early antibiotic therapy.

## Materials and methods

### Study design and setting

The patient data used in this study were obtained from adult individuals hospitalized at Zhongnan Hospital of Wuhan University with a diagnosis of CAP between 01/01/2023, and 31/12/2023. Eligible patients were classified into two groups based on the results of etiological testing and diagnoses confirmed by two senior physicians. The first group consisted of patients with MPP, defined as those infected solely with *M. pneumoniae*. The second group included patients with bacterial pneumonia, defined as those infected exclusively with bacteria and without detection of fungi, viruses, or other pathogens. Patients showing test results indicative of pathogens other than *M. pneumoniae* or bacteria were excluded from the study cohort. Additionally, immunosuppressed patients (e.g., those on glucocorticoids, undergoing radiation or chemotherapy for tumors, or co-infected with Human Immunodeficiency Virus (HIV)) were excluded. Patients’ medical data were accessed on 27/02/2024 for research purposes. This study received ethical approval from the Medical Ethics Commission of Zhongnan Hospital of Wuhan University (Approval Number: 2024038K). All data were fully anonymized prior to access. The ethics committee waived the requirement for informed consent due to the retrospective nature of the study. This study has been retrospectively registered in the Chinese Clinical Trial Registry.

### Clinical and laboratory data

Demographic and clinical characteristics, including major symptoms and signs at admission, were collected through review of the patient’s medical records. Additionally, results of the initial laboratory test conducted within 48 hours of admission, along with the conclusions of the first radiological examination and chest CT interpreted by the imaging physician, were obtained. Typically, each examination was conducted once within 48 hours of admission, and in cases of multiple tests, the results of the initial measurement were utilized. As PCT values below 0.05 ng/mL(upper reference limit) were not reported as exact numbers by our laboratory, we transformed this variable into a categorical format for analysis, and used “PCT_high_” to indicate PCT levels exceeding the upper reference limit. This approach was adopted to ensure data consistency and minimize potential bias in the results.

### Etiological diagnosis

Initial pathogenicity tests performed after patient admission include sputum culture and antibody testing for common respiratory pathogens (e.g., *influenza A and B viruses*, *human metapneumovirus*, *M. pneumoniae*, *Chlamydia pneumoniae*, etc.). Subsequently, based on the patient’s clinical manifestations, routine serological examinations (including routine blood analysis, PCT measurement, ultrasensitive C-reactive protein measurement, etc.), and imaging findings, in conjunction with diagnostic input from senior physicians, further targeted examinations like fiber optic bronchoscopy and Metagenomic next-generation sequencing (mNGS) may be considered.

Bronchoalveolar lavage fluid (BALF) samples were processed for pathogen detection using a targeted nanopore sequencing approach. For nucleic acid extraction, 400 μL of BALF was transferred into a 2 mL microcentrifuge tube containing 200 μL of 0.1 mm glass beads and subjected to mechanical disruption at 3000 rpm for 5 minutes. Following centrifugation, 300 μL of supernatant was used for DNA extraction with the TIANamp Micro DNA Kit (Tiangen Biotech, China). A Tris-EDTA buffer sample was processed in parallel as a negative extraction control. Library preparation was performed using an in-house multiplex PCR primer pool. Amplification was conducted in a 20 μL reaction system containing 5 μL of extracted DNA and 15 μL of PCR mix under the following conditions: initial denaturation at 95°C for 3 minutes; 38 cycles of 95°C for 10 seconds, 55°C for 10 seconds, and 72°C for 10 seconds; followed by a final extension at 72°C for 5 minutes. PCR products were pooled and prepared for sequencing using the Nanopore Ligation Sequencing Kit (SQK-LSK109, Oxford Nanopore Technologies, UK) and sequenced on a GridION X5 platform with an R9.4.1 flow cell. Raw signal data (FAST5 format) were basecalled and converted to FASTQ format using Guppy (v3.3.0). Low-quality reads (quality score <7) and reads with lengths <200 nt or >2000 nt were filtered out. Adapter and barcode sequences were trimmed using Porechop (v0.2.4). The remaining reads were aligned to the NCBI nucleotide (NT) database using BLASTn (v2.9.0+). Consensus sequences were generated using Medaka (v0.10.1) for species-level identification. The bioinformatics method and pathogen determination are following the previous study [17]. The criteria were used to select these pathogens as follows: (1) Filtering out closely related microorganisms [18]. (2) Filtering out contaminants from negative controls. The final report for each sample was discussed individually with a clinical microbiology specialist and clinicians responsible for each patient [19].

### Data analysis

Data analysis was conducted using IBM SPSS Statistics 27. Patients were categorized into two groups based on pathogenetic results: those with MPP and those with bacterial pneumonia. Continuous variable statistics were expressed as median and interquartile range (IQR), and discrete variable statistics were expressed as counts and percentages. Categorical variables were compared using the chi-square test or Fisher’s exact test, as appropriate. Normality was tested using the Kolmogorov–Smirnov test. For normally distributed data, the Student t-test was used to compare differences between the two groups. For non-normally distributed data, the Mann-Whitney U test was used for analysis. Univariate analyses were performed to preliminarily compare clinical and laboratory variables between the two groups and to identify potential predictors for multivariable logistic regression. Based on the aforementioned results, a binary logistic regression model was developed to identify MPP. The final model was expressed as: logit(P)=β0 + β_1_X1 + β_2_X2 + ⋯ + βkXk. In the model, P represents the predicted probability of MPP, β_0_ is the intercept, and β_1_–β_k_ are the regression coefficients for each independent variable.

### Validation

To evaluate the generalizability of the model, temporal validation was performed employing the same inclusion and exclusion criteria on a cohort of patients admitted to the same hospital between 01/01/2024 and 30/06/2024. Applying the same inclusion and exclusion criteria, we enrolled a total of 103 patients, including 52 MPP patients and 51 bacterial pneumonia patients. The area under the receiver operating characteristic (ROC) curve (AUC), sensitivity, and specificity were used to evaluate the performance of the models. Internal validation of the prediction model was performed using bootstrap resampling with 1000 iterations. Bootstrap analysis was used to evaluate the stability and robustness of the regression model and to assess the potential risk of overfitting. Decision curve analysis (DCA) was performed to evaluate the potential clinical utility of the prediction model in the validation cohort. Also, calibration performance was assessed using calibration plots.

## Results

### Baseline data

A total of 244 patients with CAP, whose infections were attributed to either *M. pneumoniae* or other bacteria, were enrolled in this study. Baseline patient characteristics are summarized in Tables 1–4 (demographics), (clinical features), (laboratory parameters), and (imaging findings). Of these, 117 patients were diagnosed with MPP, and 127 patients with other bacterial pneumonia. Among the 117 patients with confirmed MPP, the diagnostic methods used varied: 46 patients underwent *M. pneumoniae* PCR testing on nasopharyngeal/oropharyngeal swabs, 52 patients had fiber-optic bronchoscopy with alveolar lavage fluid (BALF) samples analyzed for mNGS to clarify diagnosis, and the remaining patients were diagnosed based on *M. pneumoniae*-specific antibodies in serum. In patients diagnosed with bacterial pneumonia, fiber-optic bronchoscopy was used to collect BALF for mNGS, confirming the presence of bacterial pathogens. Among these 127 bacterial pneumonia cases, *Streptococcus pneumoniae* was the most prevalent pathogen, identified in 36 patients (28.3%). Some patients also presented with multiple bacterial coinfections. Additionally, 28 had only suspected pathogenic bacteria detected in their BALF. For these patients, the true causative pathogens and final diagnoses were established by two senior physicians through comprehensive evaluation of clinical presentations, radiographic findings, and laboratory data, with consultation of clinical microbiologists [20,21].

**Table 1:** Demographics of the participants.

	*M. pneumoniae* pneumonia patients (n = 117)*,Median (IQR)*	Bacterial pneumonia patients (n = 127)*,Median (IQR)*	P-Value	AUC
Characteristics				
Age (years)	**37.00 [31.00-46.00]**	**61.00 [49.00-70.00]**	**<0.001**	**0.820**
Males, n (%)	**54(46.15)**	**78 (61.41)**		**NA**
Females, n (%)	**63(53.85)**	**49 (38.59)**		**NA**
AUC, area under the curve;				

**Table 2:** The clinical characteristics of the participants.

	*M. pneumoniae* pneumonia patients (n = 117)*,Median (IQR)*	Bacterial pneumonia patients (n = 127)*,Median (IQR)*	P-Value	AUC
Clinical characteristic n (%)				
Fever	**97 (82.91)**	**76 (59.84)**	**<0.001**	**NA**
Dry cough	**30 (25.64)**	**15 (11.81)**	**0.005**	**NA**
Nasal obstruction	**5 (4.27)**	**3 (2.36)**	**0.395**	**NA**
Myalgia	**9 (7.69)**	**3 (2.36)**	**0.054**	**NA**
Diarrhea	**2 (1.71)**	**1 (0.79)**	**0.509**	**NA**
Headaches	**20 (17.10)**	**9 (7.09)**	**0.016**	**NA**
Sore throat	**22 (18.80)**	**5 (3.94)**	**<0.001**	**NA**
Chest pain	**10 (8.55)**	**18 (14.17)**	**0.176**	**NA**
Nausea	**9 (7.69)**	**8 (6.30)**	**0.656**	**NA**
Runny nose	**3 (2.56)**	**5 (3.94)**	**0.565**	**NA**
Vital signs				
Maximum body temperature °C	**38.00 [36.80-39.00]**	**37.30 [36.60-39.00]**	**0.219**	**0.545**
Pulse (beats per minute)	**84 [78-96]**	**82 [78-88]**	**0.196**	**0.548**
Respiratory rate(beats per minute)	**20 [19-21]**	**20 [19-21]**	**0.013**	**0.412**
Serious complication, n (%)				
Respiratory Failure	**8 (6.84)**	**42 (33.07)**	**<0.001**	**NA**
AUC, area under the curve;				

**Table 3:** Laboratory parameters comparison between groups.

	*M. pneumoniae* pneumonia patients (n = 117)*,Median (IQR)*	Bacterial pneumonia patients (n = 127)*,Median (IQR)*	P-Value	AUC
Routine blood analyses				
WBC(×109/L)	**6.90 [5.40-8.17]**	**6.96 [5.26-10.18]**	**0.259**	**0.458**
NEU(×109/L)	**4.39 [3.23-5.64]**	**4.66 [3.29-7.89]**	**0.072**	**0.432**
LYM(×109/L)	**1.48 [1.04-1.91]**	**1.37 [1.00-1.80]**	**0.192**	**0.549**
MO(×109/L)	**0.62 [0.50-0.80]**	**0.60 [0.43-0.81]**	**0.399**	**0.532**
EO(×109/L)	**0.11 [0.05-0.21]**	**0.10 [0.03-0.20]**	**0.055**	**0.572**
B(×109/L)	**0.02 [0.00-0.03]**	**0.02 [0.00-0.48]**	**0.305**	**0.462**
NLR	**2.91 [2.12-4.13]**	**3.93 [2.30-5.80]**	**0.008**	**0.400**
Liver, kidney function, and electrolyte levels examination				
ALT(U/L)	**17.00 [10.75-28.00]**	**16.00 [11.00-29.00]**	**0.893**	**0.495**
AST(U/L)	**18.00 [14.00-22.25]**	**18.00 [15.00-26.00]**	**0.397**	**0.468**
TBIL(μmol/L)	**9.15 [7.18-12.08]**	**9.70 [7.13-13.55]**	**0.235**	**0.455**
ALB(g/L)	**40.68 ± 4.27**	**36.34 ± 5.76**	**<0.001**	**0.726**
GLB(g/L)	**28.61 ± 4.02**	**29.30 ± 4.30**	**0.204**	**0.458**
GGT(U/L)	**21.00 [13.00-38.50]**	**25.00 [17.00-55.75]**	**0.015**	**0.408**
BUN (mmol/L)	**3.60 [3.02-4.60]**	**4.76 [3.92-6.50]**	**<0.001**	**0.273**
CR(μmol/L)	**63.05 [56.20-79.78]**	**69.25 [56.13-81.00]**	**0.308**	**0.462**
Serum cystatin C (mg/L)	**0.89 [0.80-1.00]**	**0.97 [0.82-1.17]**	**<0.001**	**0.366**
SA (mg/L)	**810.88 ± 167.92**	**822.53 ± 203.49**	**0.632**	**0.497**
C1q(mg/L)	**209.30 [171.60-251.70]**	**169.55 [149.58-201.43]**	**<0.001**	**0.692**
K (mmol/L)	**3.83 ± 0.37**	**3.77 ± 0.42**	**0.259**	**0.546**
Na (mmol/L)	**139.45 ± 2.43**	**139.32 ± 3.36**	**0.739**	**0.520**
Blood coagulation panel				
PT (s)	**12.00 [11.50-12.50]**	**12.00 [11.40-12.90]**	**0.86**	**0.493**
INR	**1.10 [1.06-1.15]**	**1.10 [1.05-1.18]**	**0.88**	**0.494**
PTTA (s)	**85.00 [78.00-91.00]**	**86.00 [76.00-93.00]**	**0.823**	**0.492**
APTT (s)	**31.00 [29.10-32.70]**	**30.50 [28.50-32.30]**	**0.126**	**0.558**
TT (s)	**13.10 [12.50-13.90]**	**14.00 [13.40-15.00]**	**<0.001**	**0.277**
FIB (mg/dl)	**437.00 [394.00-496.00]**	**414.00 [342.50-471.00]**	**0.03**	**0.582**
Inflammation indicators				
PCT_high_, n (%)	**11 (9.40)**	**39 (30.71)**	**<0.001**	**–**
ESR(mm/h)	**23.50 [13.00-31.00]**	**28.00 [11.50-57.25]**	**0.069**	**0.429**

AUC, area under the curve; WBC, white blood cells; NEU, neutrophils; LYM, lymphocytes; MO, monocytes; EO, eosinophil; B, basophil; NLR, NEU/LYM; ALT, Alanine aminotransferase; AST, Aspartate aminotransferase; TBIL, Total bilirubin; ALB, Albumin; GLB, Globulin; GGT, γ-Glutamyl transpeptidase; BUN, urea nitrogen; CR, Creatinine; SA, sialic acid; C1q, Complement C1q; PT, Prothrombin time; INR, International normalised ratio; PTTA, Prothrombin time activity; APTT, Activated partial thromboplastin time; TT, Thrombin time; FIB, Fibrinogen; PCT, procalcitonin; PCT_high,_ PCT levels above the upper reference limit (0.05 ng/mL); ESR, haemocyte sedimentation rate;

**Table 4:** Imaging and bronchoscopy findings.

	*M. pneumoniae* pneumonia patients (n = 117), *Median (IQR)*	Bacterial pneumonia patients (n = 127)*,Median (IQR)*	P-Value	AUC
Chest CT findings n (%)				
Pleural effusion	**1 (0.85%)**	**23 (18.11%)**	**<0.001**	**NA**
Air bronchogram	**5 (4.27%)**	**2 (1.57%)**	**0.169**	**NA**

AUC, area under the curve; CT: computer tomography.

### Demographic characterization

As shown in Table 1, the univariate analysis of patient demographics revealed a significantly lower age among those with MPP compared to patients with bacterial pneumonia (37.00 years [31.00–46.00] vs. 61.00 years [49.00–70.00]). The gender distribution did not significantly differ between the two groups.

### Analysis of clinical features and ancillary examination findings

As shown in Table 2, among the clinical features, fever (82.91% vs. 59.84%, P < 0.001), dry cough (25.64% vs.11.81%, P = 0.005), and sore throat (18.80% vs. 3.94%, P < 0.001) were more prevalent in MPP patients, whereas bacterial pneumonia patients exhibited a significantly higher frequency of respiratory failure, a serious complication (6.84% vs. 33.07%, P < 0.001).

Auxiliary test results, including complete blood count, serum electrolyte levels, liver and kidney function, and coagulation function, were statistically analyzed to identify significant differences between the two groups. The concrete results were shown in Table 3.

Imaging findings showed that pleural effusion was less frequent in MPP patients than in those with bacterial pneumonia. Fiberoptic bronchoscopy revealed no significant difference between the two groups in the air bronchogram sign. The concrete results were shown in Table 4.

The complete blood count indicated that the neutrophil-to-lymphocyte ratio (NLR) (2.91 vs. 3.93, P = 0.008) was significantly lower in MPP patients compared to the bacterial pneumonia patients. Regarding liver and renal function, MPP patients showed lower levels of gamma-glutamyl transferase (GGT) (21.00 U/L vs. 25.00 U/L, P = 0.015). Similarly, renal function revealed significantly higher levels of blood urea nitrogen (BUN) (4.76 mmol/L vs.3.60 mmol/L, P < 0.001) and serum cystatin C (0.97 mg/L vs. 0.89 mg/L, P < 0.001) in the bacterial pneumonia patients compared to MPP patients, suggesting renal insufficiency.

In terms of coagulation function, as compared to patients with bacterial pneumonia, MPP patients displayed lower thrombin time (TT) (13.10 s vs. 14.00 s, P < 0.001), while prothrombin time (PT) and partially activated prothrombin time (APTT) showed no significant differences between the two groups.

### Construction of predictive model

To develop an accurate predictive model based on the differences between patient groups, variables with significant differences (*P* < 0.05) were subjected to further analysis. Indicators with an area under the curve (AUC) greater than 0.60 were considered to be included in multifactorial analyses, and the resulting independent risk factors were incorporated into the statistical models. Among indicators reflecting renal function, only urea nitrogen, with the largest AUC value, was included in the multifactorial regression analysis. A similar method was conducted on ALB and ALB/GLB. Ultimately, 6 indicators were selected for subsequent multifactorial regression analysis: age, serum albumin, urea nitrogen, complement C1q levels, procalcitonin, and TT.

Based on the results of the multifactorial logistic regression analysis, five indicators – age, ALB, complement C1q, PCT_high_, and TT were identified as the elements for constructing the prediction model (e.g., Table 5). After adjusting for other variables, BUN (P = 0.839) was not statistically significant and thus was excluded from the model construction. The resulting model can be expressed as follows: Logit(P)=−0.167 − 0.063 × Age + 0.106 × ALB + 0.014 × C1q−1.239 × PCT_high_−0.360 × TT (P, predicted value; e, natural logarithm). A higher value obtained from this formula indicates an increased probability of distinguishing the two types of pneumonia patients.

**Table 5:** Univariate and multivariate analyses of makers between two groups.

Variable	Univariate analysis			Multivariate analysis		
	OR	95%CI	P-Value	OR	95%CI	P-Value
ALB (g/L)	1.184	1.116-1.255	<0.001	1.112	1.026-1.206	0.010
Age (years)	0.919	0.900-0.939	<0.001	0.939	0.915-0.964	<0.001
C1q (mg/L)	1.014	1.008-1.020	<0.001	1.014	1.007-1.022	<0.001
PCT_high_, n (%)	0.243	0.118-0.504	<0.001	0.290	0.107-0.786	0.015
BUN(mmol/L)	0.730	0.622-0.858	<0.001	1.014	0.890-1.155	0.839
TT	0.533	0.418-0.630	<0.001	0.698	0.512-0.950	0.022

C1q, Complement C1q; PCT, procalcitonin; BUN, urea nitrogen; TT, Thrombin time; ALB, Albumin; PCT_high,_ PCT levels above the upper reference limit (0.05 ng/mL)

### Comparison of predictive modeling with single indicator predictive value

This statistical model, comprising five indicators including age, serum albumin, complement C1q, procalcitonin, and thrombin time, significantly identifies MPP patients between the two types of pneumonia patients, achieving an AUC value of 0.879 (95% CI, 0.833–0.925) (Fig 1). The Youden-index optimized cutoff was 0.469, demonstrating ideal diagnostic performance (sensitivity 85.7%, specificity 81.7%). To compare the model’s discriminatory ability between *M. pneumoniae* and bacterial pneumonia, its test efficacy was compared to models constructed with single independent risk factors, as previously reported (Table 6). For the model based solely on age as a variable, the calculated AUC value was 0.820, yielding a sensitivity of 78.7% and specificity of 76.9% at a cutoff age of 47.5 years. Likewise, predictive models using serum albumin level and complement C1q level are detailed in Table 6.

**Table 6:** Performance of the single- and five-marker models.

Variable			Value (95%CI)		
	ALB (g/L)	AGE (years)	C1q (mg/L)	TT (s)	MODEL
AUC	0.726 [0.662-0.790]	0.820 [0.766-0.874]	0.692 [0.625-0.759]	0.723 [0.658-0.789]	0.879 [0.833-0.925]
Cut-off	37.05	47.5	203.75	13.35	0.469
Sensitivity(%)	80.7	78.7	55.8	77.6	85.7
Specificity(%)	58.9	76.9	76.0	61.3	81.7
Accuracy(%)	66.8	77.9	66.0	68.2	82.7

AUC, area under the curve; C1q, Complement C1q; TT, Thrombin time; ALB, Albumin

**Fig 1:**
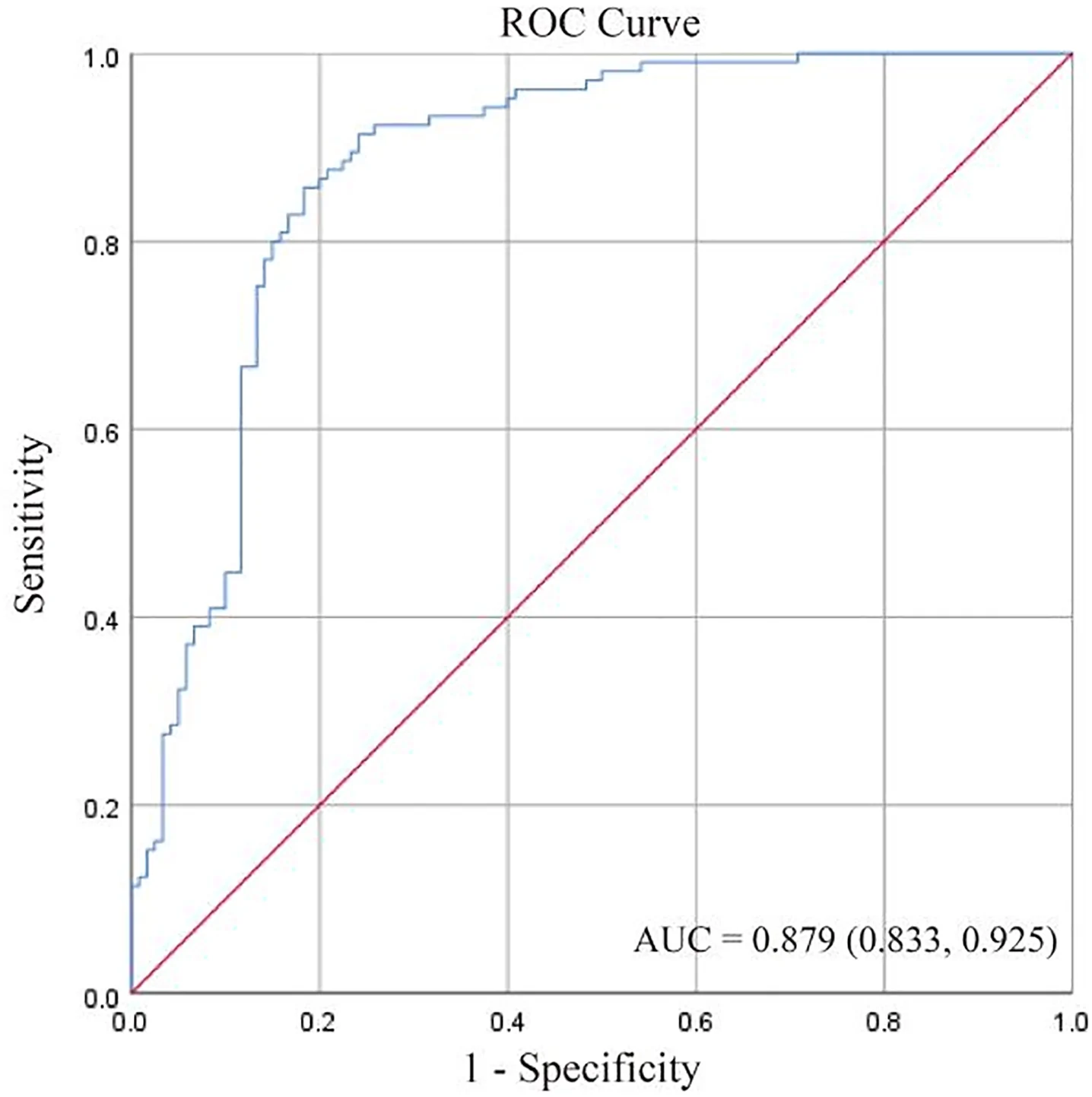
ROC curve of the prediction model, derivation cohort. AUC, area under the receiver operating characteristic curve; ROC, receiver operating characteristic.

### Simplified model

To evaluate the applicability of the model in settings where complement C1q testing is not routinely available, we further developed a simplified model excluding C1q. The resulting model can be expressed as follows: Logit(P)=4.343 − 0.074 × Age + 0.077 × ALB − 1.239 × PCT_high_−0.353 × TT (P, predicted value; e, natural logarithm). The Youden-index optimized cutoff was 0.386. In the derivation cohort, the simplified model achieved an accuracy of 82.4%, with a sensitivity of 91.4% and a specificity of 77.9% (Table 7).

**Table 7:** Performance of the simplified model.

AUC	Cut-off	Sensitivity(%)	Specificity(%)	Accuracy(%)
0.856 (0.805-0.908)	0.386	91.4%	77.9%	82.4%

AUC, area under the curve.

### Validation results

#### Comparison of baseline characteristics between derivation and validation cohorts.

Baseline characteristics of the derivation cohort (n = 244) and validation cohort (n = 103) are summarized in Table 8 below. The two cohorts showed no significant differences in the five variables included in the final model (all P > 0.05): age, albumin (ALB), complement C1q (C1q), thrombin time (TT), and PCT.

**Table 8:** Baseline characteristics of derivation and validation cohorts.

Characteristic	Derivation Cohort (n = 244)	Validation Cohort (n = 103)	P-value
**ALB(g/L)**	**38.40 ± 5.62**	**37.54 ± 5.15**	**0.160**
**Age (years)**	**50.00 [35.00-62.00]**	**51.00 [36.00-62.00]**	**0.527**
**C1q (mg/L)**	**188.15 [155.60-228.78]**	**193.10 [162.80-231.60]**	**0.193**
**TT (s)**	**13.60 [12.83-14.70]**	**13.10 [12.30-14.20]**	**0.190**
**PCT**_**high**_ **, n (%)**	**50(20.49)**	**24 (23.30)**	**0.611**

AUC, area under the curve; C1q, Complement C1q; TT, Thrombin time; ALB, Albumin; PCT_high,_ PCT levels above the upper reference limit (0.05 ng/mL)

Given that no significant differences were observed in baseline characteristics between the two groups, we utilized this dataset as a temporal validation cohort to evaluate the model. The model achieved an AUC of 0.922 (95% CI: 0.871–0.973) in the validation cohort, demonstrating a sensitivity of 92.3% and a specificity of 80.4%. Furthermore, the Hosmer-Lemeshow test yielded a P-value of 0.47 (>0.05), indicating good predictive performance and stability of the model. Detailed results are presented in Table 9 and Fig 2. Calibration analysis demonstrated acceptable agreement between predicted and observed probabilities in the derivation and validation cohorts. The corresponding calibration plots are provided in the Supplementary Material.

**Table 9:** Comparison of the derivation cohort and the validation cohort.

Characteristic	Derivation Cohort (n = 244)	Validation Cohort (n = 103)
**AUC (95% CI)**	**0.879 [0.833-0.925]**	**0.922 [0.871-0.973]**
**Sensitivity**	**85.7%**	**92.3%**
**Specificity**	**81.7%**	**80.4%**

AUC, area under the curve.

**Fig 2:**
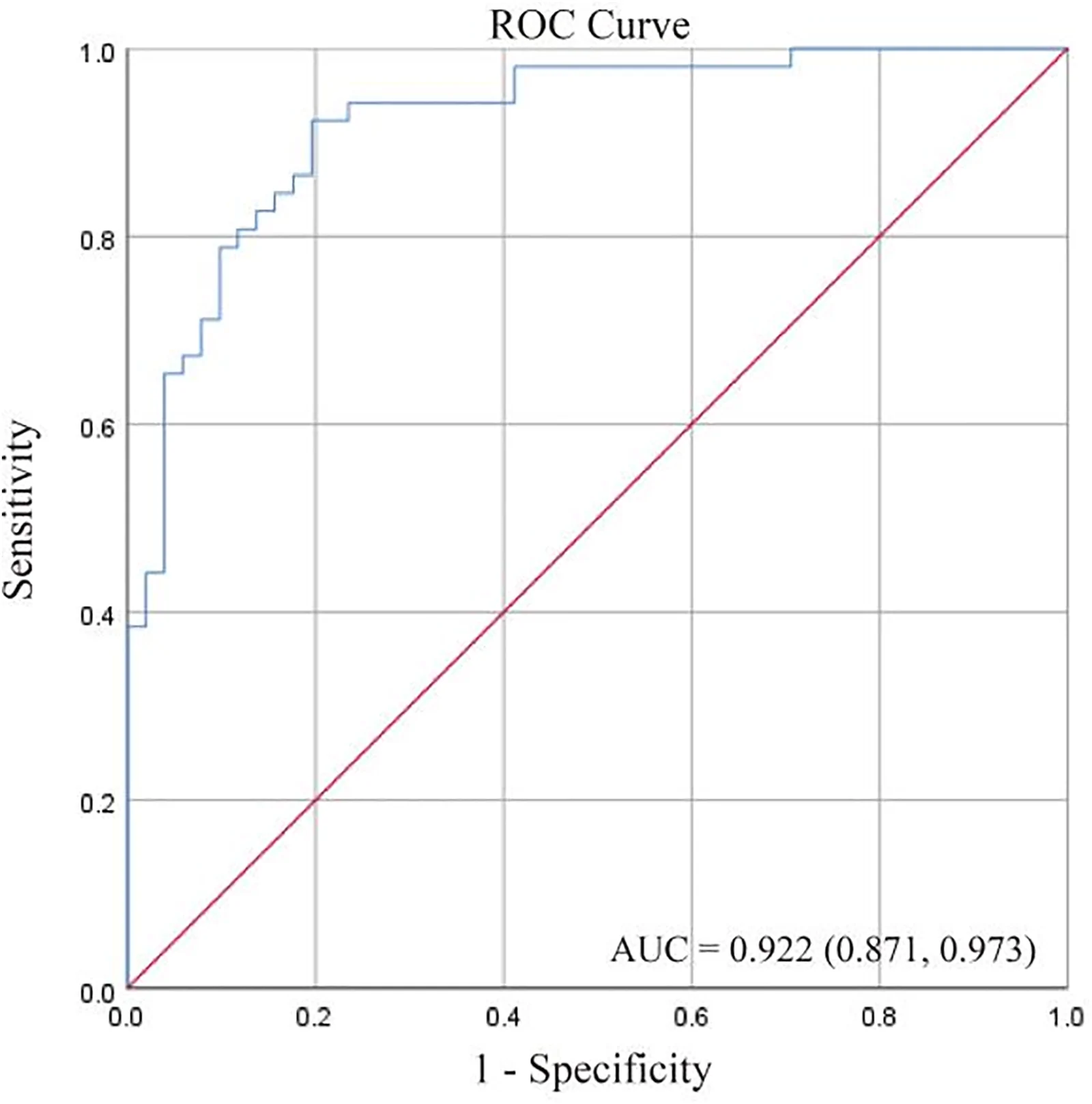
ROC curve of the prediction model, validation cohort. AUC, area under the receiver operating characteristic curve; ROC, receiver operating characteristic.

Bootstrap internal validation was performed using 1000 resamples. The regression coefficients of all predictors remained stable across bootstrap samples, and all variables retained statistical significance after resampling. The bootstrap results demonstrated good model robustness with no evidence of substantial overfitting, supporting the internal validity of the prediction model.

Decision curve analysis demonstrated that the prediction model provided a higher net benefit than both the treat-all and treat-none strategies across a wide range of threshold probabilities in the derivation and validation cohort (Fig 3 and 4), indicating potential clinical utility.

**Fig 3:**
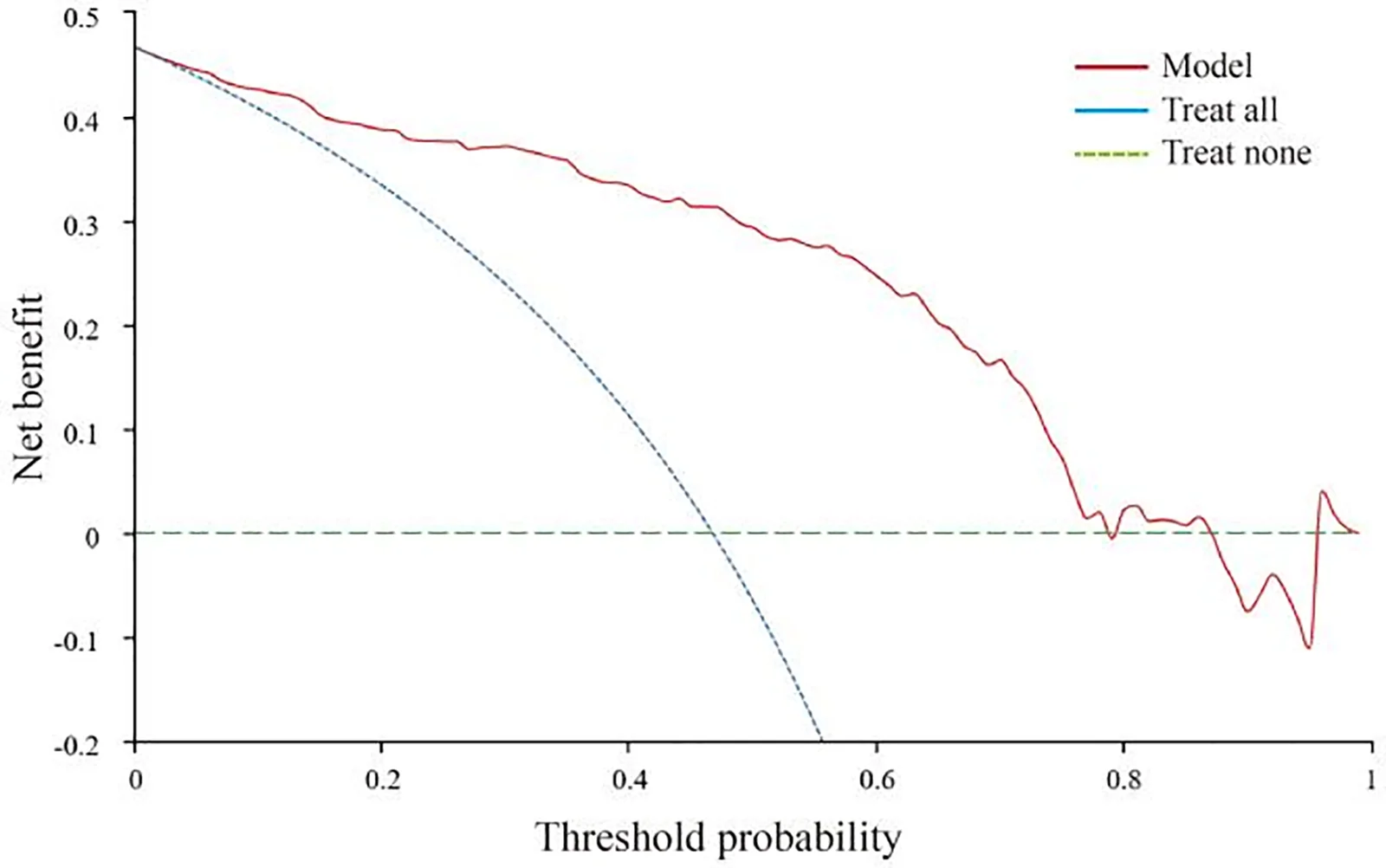
Decision curve analysis, derivation cohort.

**Fig 4:**
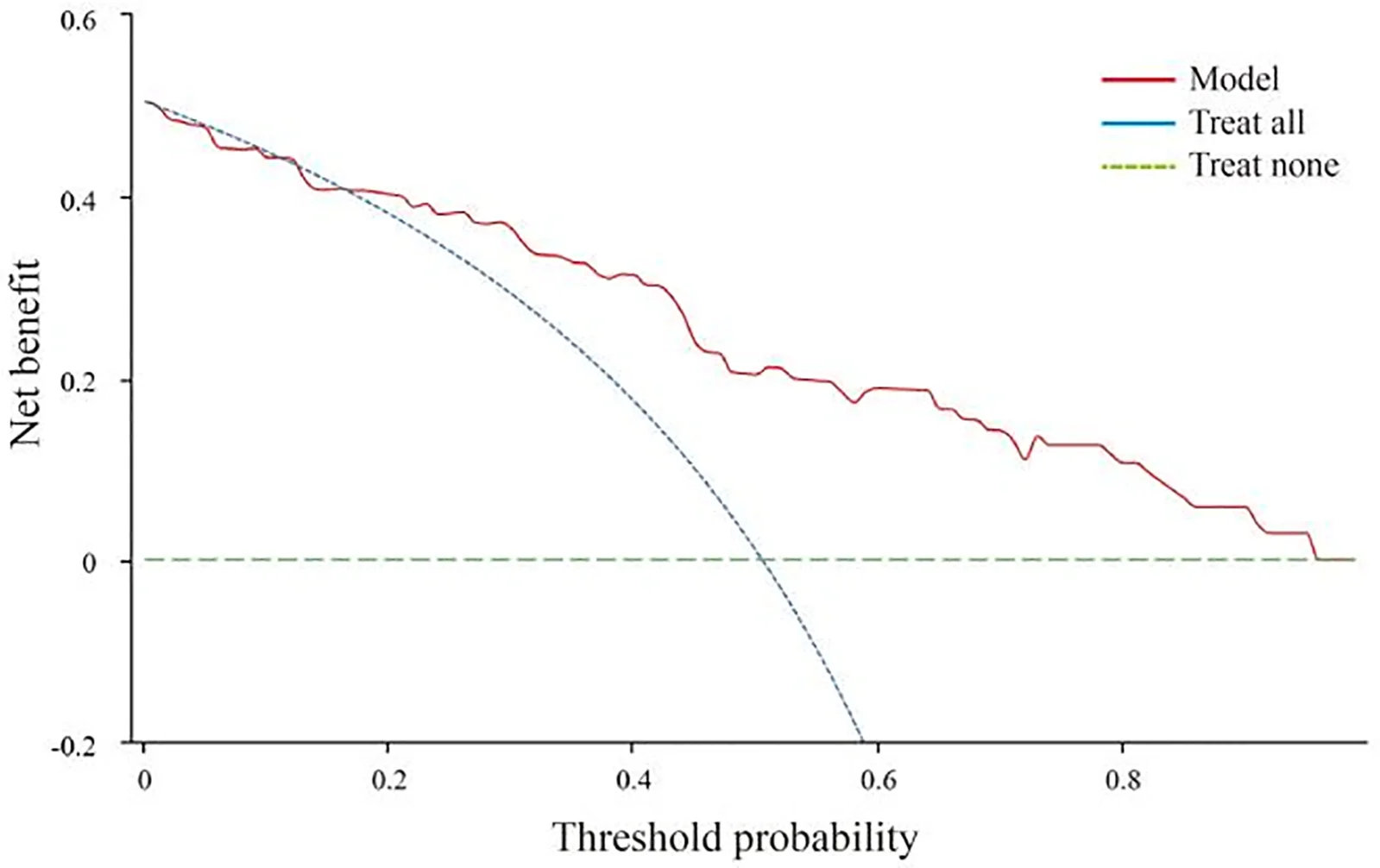
Decision curve analysis, validation cohort.

Using the same method, we evaluated the simplified model in the same validation cohort. The simplified model achieved an AUC of 0.925 (95% CI: 0.872–0.976) in the validation cohort, with a sensitivity of 88.5% and a specificity of 88.2%. In addition, the Hosmer–Lemeshow test yielded a P-value of 0.457 (>0.05), indicating good predictive performance and stability of the model. Detailed results are presented in Table 10. Calibration analysis demonstrated acceptable agreement between predicted and observed probabilities in both the derivation and validation cohorts, with the corresponding calibration plots provided in the Supplementary Material (S1 and S2 Figs).

**Table 10:** Comparison of the simplified model between the derivation cohort and the validation cohort.

Characteristic	Derivation Cohort (n = 244)	Validation Cohort (n = 103)
**AUC (95% CI)**	**0.856 [0.805-0.908]**	**0.925 [0.872-0.976]**
**Sensitivity**	**91.4%**	**88.5%**
**Specificity**	**77.9%**	**88.2%**

AUC, area under the curve.

## Discussion

Distinguishing *Mycoplasma pneumoniae* pneumonia (MPP) from bacterial pneumonia in the early stages presents a common clinical challenge that significantly affects the selection and efficacy of empirical antibiotic treatment. To address this issue, we conducted a case-control study comprising 244 patients and then successfully developed a novel prediction model for identifying MPP patients. The multivariate analysis identified five clinically accessible biomarkers – age, albumin, complement C1q, thrombin time, and procalcitonin – as independent predictors of adult MPP. The robust performance of our model (AUC 0.879, sensitivity 85.7%, specificity 81.7%) suggests its potential as a practical clinical tool for MPP identification. Subsequent validation further confirmed the model’s strong performance.

In our research, younger age emerged as a strong predictor of MPP, corroborating previous reports [22]. Furthermore, the established relationship between hypoalbuminemia and elevated inflammatory markers (including C-reactive protein) in prior research supports albumin’s potential role as a supplementary biomarker for pathogen classification in CAP [23]. Similar studies have indicated that decreased levels of albumin may serve as a biomarker for disease severity and prognostic evaluation [24,25]. These findings are consistent with our results that compared with bacterial pneumonia, higher ALB levels in MPP patients, as observed in this study, may reflect relatively preserved nutritional status and milder systemic inflammation.

Procalcitonin, a well-established clinical biomarker, is routinely utilized in clinical practice and has been validated as a reliable biomarker for both diagnosing bacterial infections and guiding antibiotic therapy [26–28]. In our study, a significantly higher proportion of bacterial pneumonia patients exhibited procalcitonin levels above the reference limit (>0.05 ng/mL) compared to MPP, consistent with previous research [29]. This outcome further supports the reliability of Procalcitonin as a preliminary screening parameter for suspected MPP cases.

Moreover, we observed that in our cohort, serum complement C1q levels and thrombin time (TT) differed between patients with *M. pneumoniae* pneumonia and those with bacterial pneumonia. As for the former, it has established roles in both innate and adaptive immunity [30]. C1q has been implicated in the regulation of neutrophil extracellular traps (NETs) and their interaction with coagulation pathways, which may contribute to host immune responses during infection [31].Similarly, as for TT, previous studies have reported that bacterial infections, particularly in severe pneumonia or sepsis, often trigger systemic inflammatory responses leading to coagulation abnormalities, including consumption of coagulation factors and prolongation of TT [32]. However, these observations remain largely associative, the underlying mechanisms require further elucidation.

Except for the individual predictive indicators, it is important to consider the clinical applicability and potential impact of our model. The integration of readily available clinical and laboratory parameters into a predictive model offers a pragmatic approach for early prediction of MPP. Our model demonstrated high sensitivity (85.7%), effectively reducing the missed diagnosis rate of MPP. Furthermore, the model’s performance in temporal validation (AUC 0.922) underscores its robustness and generalizability, suggesting its potential for broader adoption. Notably, a simplified model still demonstrated good predictive performance, suggesting that the model may be adapted for use in resource-limited environments. Current non-invasive diagnostic methods for MPP, including nucleic acid amplification and IgM antibody assays, show suboptimal sensitivity [33], and their availability remains limited in some primary healthcare settings. The predictive model developed in this study, based on readily accessible clinical and laboratory indicators, may therefore provide an alternative approach for early recognition of MPP in hospitals where such specialized tests are not routinely available.

Our study has several limitations. First, the pathogenicity test, fiberoptic bronchoscopy with alveolar lavage, is typically performed one to two days after patient admission. As a result, the empirical anti-infective treatment administered post-admission may affect the detection of pathogen species in the alveolar lavage fluid, making it challenging to accurately represent the initial pathogen profile. Second, as a single-center, retrospective study, the generalizability of our findings may be limited by the sample size, and the analysis would benefit from a larger and more diverse cohort in future studies. Lastly, the occurrence of mixed infections and limitations in detection methods mirrors clinical complexity. Future studies with a larger inclusion of diverse pathogen profiles are warranted to validate the model.

## Conclusion

Our findings identified several key indicators, including younger age, elevated serum albumin and complement C1q levels, normal procalcitonin levels, and shorter thrombin time, that are strongly associated with MPP. Using these indicators, we developed a predictive model to identify MPP patients and thereby facilitating early antibiotic selection and clinical decision-making.

## Supporting information

S1 Fig Calibration curve of the model, derivation cohort. (JPG)

S2 Fig Calibration curve of the model, validation cohort. (JPG)

## Abbreviations

CAP: Community-Acquired Pneumonia
ALB: Albumin
PCT: Procalcitonin
NEU: Neutrophil
LYM: Lymphocyte
WBC: White Blood Cells
M: Monocytes
EOS: Eosinophils
RBC: Red Blood Cells
PLT: Platelet
Hb: Hemoglobin
ALT: Alanine Aminotransferase
AST: Aspartate Aminotransferase
TBIL: Total Bilirubin
GLB: Globulin
ALB/GLB: Albumin/Globulin
SA: Serum Amyloid
CR: Creatinine
PT: Prothrombin Time
INR: International Normalized Ratio
PTTA: Prothrombin Time Activity
APTT: Activated Partial Thromboplastin Time
TT: Thrombin Time
FIB: Fibrinogen
AUC: Area Under Curve
SD: Standard Deviation
ROC: Receiver Operating Characteristic
PPV: Positive Predictive Value
NPV: Negative Predictive Value
CI: Confidence Intervals
IQR: Interquartile Range
NETs: Neutrophil Extracellular Traps
mNGS: Metagenomic next-generation sequencing
